# Unilateral retinocytoma associated with a variant in the *RB1* gene

**DOI:** 10.1002/mgg3.1156

**Published:** 2020-01-29

**Authors:** Shijing Wu, Xuan Zou, Zixi Sun, Tian Zhu, Xing Wei, Ruifang Sui

**Affiliations:** ^1^ Department of Ophthalmology Peking Union Medical College Hospital Peking Union Medical College and Chinese Academy of Medical Sciences Beijing China

**Keywords:** *RB1* gene, retinoblastoma, retinocytoma

## Abstract

**Background:**

Retinocytoma is a rare benign retinal tumor associated with variants in the *RB1* gene. Ophthalmoscopic features can include a translucent retinal mass, calcification, retinal pigment epithelial alteration and chorioretinal atrophy.

**Methods:**

Detailed ophthalmological examinations were performed in a Chinese patient with retinocytoma and his daughter with bilateral retinoblastoma. Sanger sequencing was performed to detect *RB1* genetic variants in the patient, his daughter and tumor tissue from his daughter.

**Results:**

A 33‐year‐old man presented with poor vision and strabismus in the right eye since childhood. Fundus examination revealed a round yellow‐white lesion stretching from the nasal side of the optic disc to the temporal periphery of the right eye. Sequencing result identified a reported variant (c.658C>G, p.Leu220Val) in the *RB1* gene (NM_000321.2) of DNA extracted from peripheral blood of the patient and his daughter. The missense variant was also found in the tumor tissue from his daughter.

**Conclusions:**

We report detailed clinical features and genetic analysis of a case with unilateral retinocytoma. Retinocytoma has a wide range of clinical phenotypes; genetic testing is therefore a useful tool for the diagnosis of atypical cases.

## Graphical abstract

Retinocytoma is a rare benign retinal tumor associated with variants in the RB1 gene. Retinocytoma has a wide‐range of clinical phenotypes; genetic testing is therefore a useful tool for the diagnosis of atypical cases.

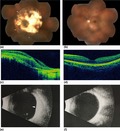

## 1 INTRODUCTION

Retinocytoma is a rare, benign retinal tumor, first described by Gallie, Ellsworth, Abramson, and Phillips (1982); its incidence in the general population is unknown. Histopathologic study has shown that the tumor consists entirely of benign‐appearing mature retinal cells without necrosis or mitoses (Margo, Hidayat, Kopelman, & Zimmerman, 1983). *RB1* gene (OMIM 614041) variants can be found in patients with retinocytomas (Abramson, 1983). Ophthalmoscopic features of retinocytoma include a translucent retinal mass, calcification, retinal pigment epithelial alteration and chorioretinal atrophy (Singh, Santos, Shields, Shields, & Eagle, 2000). Because retinocytoma and retinoblastoma appear similar, other terms such as spontaneously regressed retinoblastoma, spontaneously arrested retinoblastoma, and retinoblastoma group 0 have also been used to describe retinocytoma (Aaby, Price, & Zakov, 1983; Abramson, 1983). Here, we present a case of unilateral retinocytoma with detailed clinical ocular examinations, who carried a missense variant (c.658C>G, p.Leu220Val) in the *RB1* gene (NM_000321.2).

## 2 CASE REPORT

**Figure 1:**
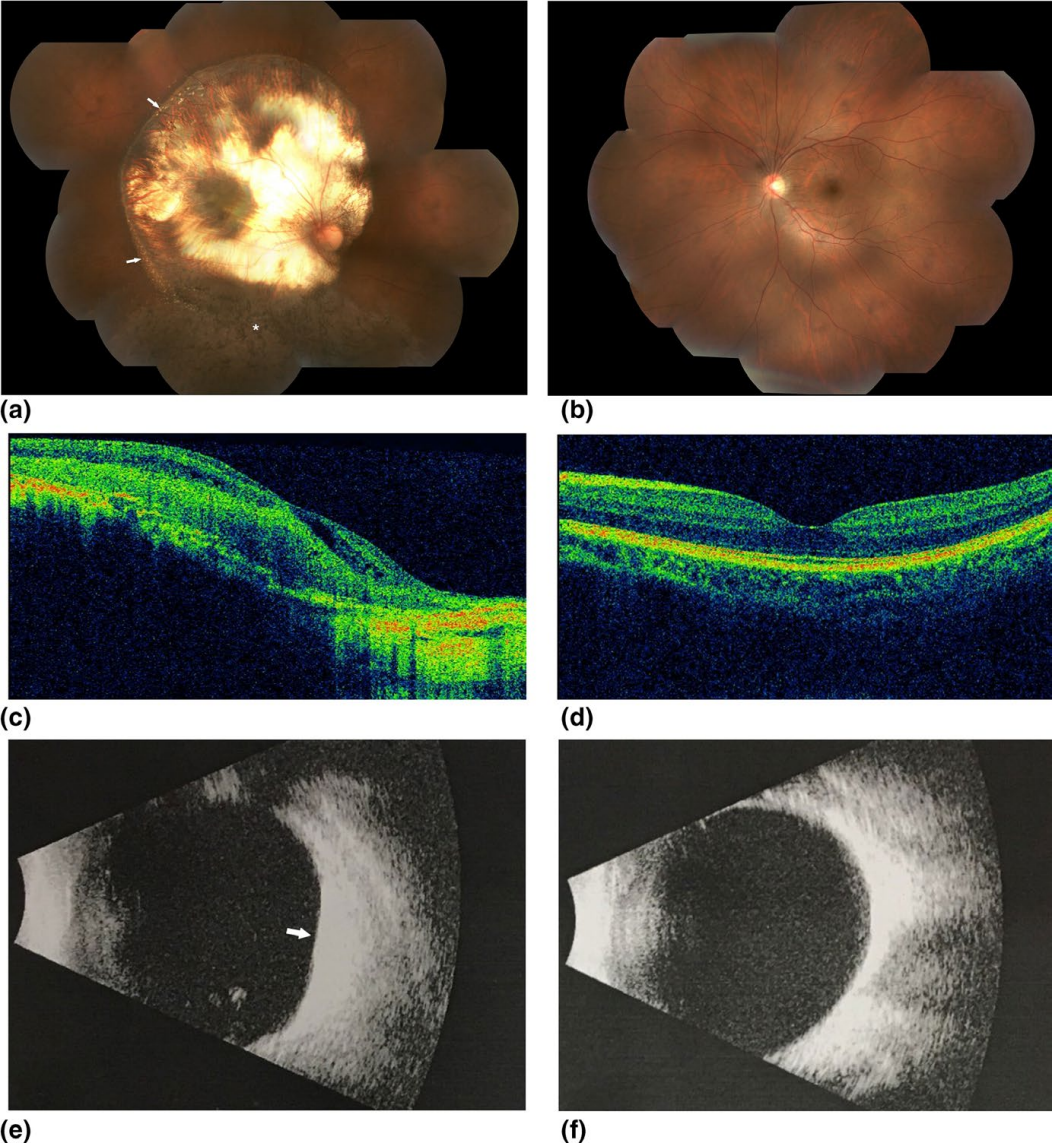
Fundus images of the patient. Color fundus photograph of right eye shows a large round‐shaped mildly elevated lesion with irregular yellowish‐white patches (arrows). The lesion is surrounded by chorioretinal and retinal pigment epithelial atrophy superior‐temporally and pigment proliferation (asterisk) in the inferior retina (a). The left fundus is normal (b).OCT images showing hyper‐reflective and thickened outer retina layers and thinned inner retina layers in right eye (c).OCT of the left eye is normal (d). B‐scan ultrasonography demonstrating a thick hyper‐reflective lesion (arrows) in right eye (e) and normal left eye (f)

**Figure 2:**
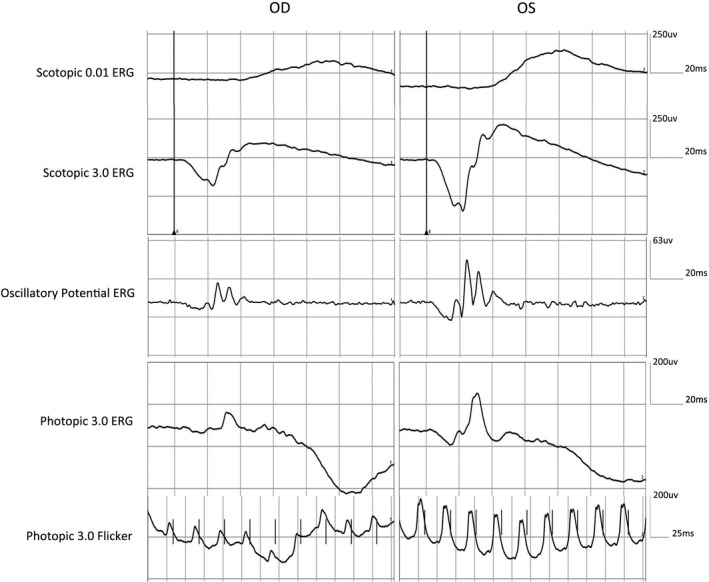
ERG recordings of the patient. Mildly reduced rod and cone responses in right eye and normal responses in left eye were recorded

A 33‐year‐old man presented to the Department of Ophthalmology, Peking Union Medical College Hospital because of poor vision in his right eye. Visual acuity of right eye had been poor since childhood and had not changed significantly over the years. He was initially diagnosed with chorioretinal atrophy. At age 26, his right eye underwent strabismus surgery in another hospital without improvement of vision. He had no systemic disease or history of trauma to either eye. His best‐corrected visual acuity was hand motion in the right eye and 20/20 in the left eye. The anterior segment slit‐lamp examination and the intraocular pressure were normal in both eyes. Fundus examination of the right eye revealed a large, round‐shaped mildly elevated lesion with irregular yellowish‐white patches surrounded by retinal pigment epithelial (RPE) atrophy in the superior and temporal border, and pigment proliferation inferiorly (Figure 1a). Optical coherence tomography (OCT; Topcon) showed hyper‐reflective and clearly thickened outer neuroretina layers (Figure 1c). Consistent with the OCT result, B‐scan ultrasonography (Aviso A/B, Quantel Medical) also suggested thickening of the retina (Figure 1e). In order to evaluate retinal function, a standard full field electroretinogram (ERG; RetiPort ERG system; Roland Consult) was performed and showed mildly reduced responses from right eye (Figure 2). All examinations of his left eye were normal. (Figures 1b,d,f, 2). We continued to inquire about his family history. His daughter was diagnosed with retinoblastoma in both eyes at 6 months of age and her left eye had undergone enucleation with pathological confirmation. Given these results and the family history, a diagnosis of retinocytoma was made in the father's case. No treatment was offered other than close observation. The size of the lesion and the visual acuity has remained unchanged at 1 year of follow‐up.

## 3 GENETIC ANALYSIS

This study adhered to the tenets of the Declaration of Helsinki and was approved by the Ethics Committee of Peking Union Medical College Hospital. Written informed consent was obtained from the patient. Blood samples were collected from the patient and his daughter, and formalin‐fixed paraffin embedded (FFPE) tumor tissue from child. DNA extraction was performed with a QIAamp DNA Blood Midi Kit and a QIAamp DNA FFPE kit (Qiagen) according to the manufacturer's protocol. Polymerase chain reactions (PCR) were designed to amplify *RB1* exons and splice‐sites. After purification, amplicons were sequenced using both forward and reverse primers on an ABI 3730 Genetic Analyzer (ABI). The results were compared with an *RB1* reference sequence (NM_000321.2). Sanger sequencing confirmed a reported variant (c.658C>G, p.Leu220Val) in the peripheral blood of the father and his daughter. This missense variant was also found in the tumor tissue from his daughter.

## 4 DISCUSSION

Retinocytoma is easily misdiagnosed. Most patients are diagnosed with retinocytoma when they come for an eye examination when their family members are diagnosed with retinoblastoma, especially first‐degree relatives. The diagnosis can also be made in symptomatic cases based on symptoms of visual loss or strabismus (Yaman, Gunduz, Saatci, & Kocak, 2008). Retinocytoma may appear similar to retinoblastoma. In previous studies, many researchers have reported how to differentiate these two conditions (Gallie et al., 1982; Margo et al., 1983). Retinocytoma has a wide‐range of clinical phenotypes. Typical symptoms and a positive family history are key points for diagnosis, but only 10% of cases present with all four diagnostic, ophthalmoscopic features: a translucent retinal mass (88%), calcification (63%), retinal pigment epithelial alteration (54%), and chorioretinal atrophy (54%) (Singh et al., 2000). In our case, the patient had poor vision since childhood, but wasn't diagnosed accurately until his daughter was found to have retinoblastoma. The lesion should be differentiated from other chorioretinal diseases, for example, chorioretinal atrophy or coloboma. There are no previous reports of ERG testing in retinocytoma patients. Our patient showed mildly reduced responses from the right eye, indicating that the function of patient's peripheral retina was preserved.

Retinocytoma and retinoblastoma can arise in the same patient. Some patients may have retinoblastoma in one eye and retinocytoma in the fellow eye, as two separate foci (Balmer, Munier, & Gailloud, 1991; Gallie et al., 1982). Although we generally believe that retinocytoma is a nonprogressive and benign retinal tumor, 4%–12% of cases may undergo malignant transformation into retinoblastoma (Abouzeid et al., 2012; Singh et al., 2000). As in all published reports, the sample size was relatively small, transformation rates to malignancy have to be interpreted with caution (Abouzeid et al., 2012). Abouzeid et al. (2012) reviewed the outcomes of 36 cases of retinocytoma and found one case with a non‐ocular neoplasm (canalicular breast cancer). Korswagen, Moll, Imhof, and Schouten‐van Meeteren (2004) have also postulated that the risk of developing another primary tumor might be increased for patients with retinocytoma. Therefore, regular annual follow‐up is suggested.

The *RB1* gene, located at 13q14, was the first tumor suppressor gene cloned (Friend et al., 1986). A retinoblastoma develops according to the “two‐hit” model with both alleles involved. The first “hit” is usually in the germline, and inherited from either parent or acquired de novo in the early stages of embryo development. The second “hit” is a somatic variant after the zygote is formed (Harbour & Dean, 2000). Several theoretical mechanisms have been proposed to explain the formation of a retinocytoma. Retinocytoma could occur if the second “hit” happens at a latest age of cell maturation when the precursor cell has limited mitotic capability and can't accumulate extragenetic variants that might predispose the tumor to grow (Gallie, Dunn, Chan, Hamel, & Phillips, 1991). Retinocytoma could also be a manifestation of low‐penetrance retinoblastoma (Dryja, Rapaport, McGee, Nork, & Schwartz, 1993; Harbour, 2001; Kratzke et al., 1994).It is possible that proteins encoded by *RB1* gene variants retain part of the wild‐type protein function (Lohmann, Brandt, Hopping, Passarge, & Horsthemke, 1994). Therefore, in the presence of a partially functional RB1 protein, the precursor cells form a retinocytoma instead of a retinoblastoma (Harbour, 2001; Lohmann et al., 1994; Otterson, Chen, Coxon, Khleif, & Kaye, 1997; Sakai, Ohtani, McGee, Robbins, & Dryja, 1991).

In conclusion, we describe the ocular manifestations of a unilateral retinocytoma in a patient with a missense variant in the *RB1* gene which expands the genotypic and phenotypic spectrum of retinocytoma. Retinocytoma can occur in childhood without other remarkable ocular manifestations and genetic testing is a useful tool for the diagnosis of atypical cases. Regular annual follow‐up visits are therefore recommended for retinocytoma patients.

## CONFLICT OF INTEREST

The authors have no disclosures or other conflicts of interest to report.

## AUTHOR CONTRIBUTION

SW wrote the manuscript. XW performed the Sanger sequencing. XZ, ZS and TZ examined the patients. RS co‐ordinated the care of the patient and his investigations.
